# Retrospective Study of Reported Adverse Events Due to Complementary Health Products in Singapore From 2010 to 2016

**DOI:** 10.3389/fmed.2018.00167

**Published:** 2018-06-12

**Authors:** Yimin Xu, Dhavalkumar N. Patel, Suet-Leng P. Ng, Siew-Har Tan, Dorothy Toh, Jalene Poh, Adena Theen Lim, Cheng-Leng Chan, Min-Yong Low, Hwee-Ling Koh

**Affiliations:** ^1^Department of Pharmacy, Faculty of Science, National University of Singapore, Singapore, Singapore; ^2^Applied Sciences Group, Pharmaceutical Division, Health Sciences Authority, Singapore, Singapore; ^3^Vigilance, Compliance and Enforcement Cluster, Health Products Regulation Group, Health Sciences Authority, Singapore, Singapore

**Keywords:** complementary health products, adverse events, pharmacovigilance, glucosamine, adulterants

## Abstract

The objective of this study is to collate and analyse adverse event reports associated with the use of complementary health products (CHP) submitted to the Health Sciences Authority (HSA) of Singapore for the period 2010–2016 to identify various trends and signals for pharmacovigilance purposes. A total of 147,215 adverse event reports suspected to be associated with pharmaceutical products and CHP were received by HSA between 2010 and 2016. Of these, 143,191 (97.3%) were associated with chemical drugs, 1,807 (1.2%) with vaccines, 1,324 (0.9%) with biological drugs (biologics), and 893 (0.6%) with CHP. The number of adverse event reports associated with Chinese Proprietary Medicine, other complementary medicine and health supplements are presented. Eight hundred and ninety three adverse event reports associated with CHP in the 7-year period have been successfully collated and analyzed. In agreement with other studies, adverse events related to the “skin and appendages disorders” were the most commonly reported. Most of the cases involved dermal allergies (e.g., rashes) associated with the use of glucosamine products and most of the adulterated products were associated with the illegal addition of undeclared drugs for pain relief. Dexamethasone, chlorpheniramine, and piroxicam were the most common adulterants detected. Reporting suspected adverse events is strongly encouraged even if the causality is not confirmed because any signs of clustering will allow rapid regulatory actions to be taken. The findings from this study help to create greater awareness on the health risks, albeit low, when consuming CHP and dispelling the common misconception that “natural” means “safe.” In particular, healthcare professionals and the general public should be aware of potential adulteration of CHP. The analysis of spontaneously reported adverse events is an important surveillance system in monitoring the safety of CHP and helps in the understanding of the risk associated with the use of such products. Greater collaboration and communication between healthcare professionals, regulators, patients, manufacturers, researchers, and the general public are important to ensure the quality and safety of CHP.

## Introduction

**Figure 1:**
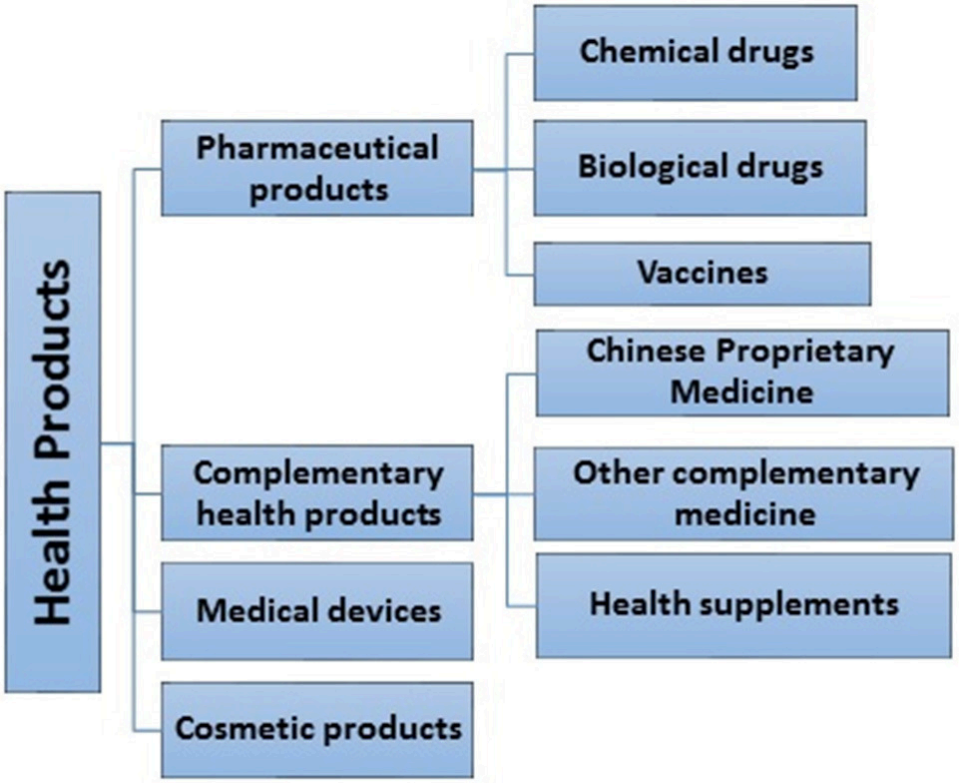
Types of health products.

In Singapore, health products are defined as any substance, preparation or device intended for use by humans, solely or principally for a health-related purpose. These include pharmaceutical products, complementary health products (CHP), medical devices, and cosmetic products (Figure 1). CHP consists of Chinese proprietary medicines (CPM), traditional medicines (TM), homeopathic medicines, health supplements (HS), and traditional medicinal materials (1). There have been some concerns about the safety of CHP, in particular adverse effects associated with them. World Health Organization (WHO) defines adverse drug reaction as a reaction which is noxious and unintended, and which occurs at doses normally used in man for prophylaxis, diagnosis, or therapy of a disease, or for the modification of a physiological function (2). An adverse event is “a medical occurrence temporally associated with the use of a medicinal product, but not necessarily causally related” (2). For the purpose of this paper, the term “adverse event” will be used.

Although CHP are widely perceived to be safe by consumers on the basis of their natural origin and long term use by various cultures, factors such as poor quality, incorrect or misidentified herbs, incorrect processing methods, variations in the concentration of active ingredients in products, inherent toxicity of herbs (e.g., hepatotoxicity, nephrotoxicity), contamination or deliberate adulteration with harmful substances or synthetic drugs, herb-herb interactions, and herb-drug interactions can affect the quality and safety of these products (3–6).

Regulation of CHP in many developed countries (including Singapore) focuses primarily on post-market strategies like post-market surveillance testing and adverse event monitoring. Recognizing the public health issues and concerns surrounding the use of herbal medicines, the “WHO Traditional Medicine Strategy 2014–2023” was established. The strategy aims to support Member States in developing proactive policies and implementing action plans for monitoring of herbal safety within the existing pharmacovigilance framework (7). Pharmacovigilance has great significance in detecting adverse effects as many herbal products on the market have not been thoroughly tested for their efficacy and safety.

Different methods are used for pharmacovigilance of medicines including spontaneous reporting, prescription event monitoring and case control, and cohort studies (8). As these methods are primarily developed for conventional drugs, they are currently of little use for evaluating the safety of herbal medicines although modified methods have been developed. Spontaneous reporting of adverse events has played a major role in post-market drug surveillance and has led to the withdrawal of pharmaceutical products as well as herbal medicinal products such as Kava kava. Therefore, international and national databases of reported adverse events can serve as an effective tool to identify signals and trends of adverse events associated with the use of herbal medicinal products (9).

The objective of this study is to collate and analyse adverse event reports associated with the use of CHP submitted to the Health Sciences Authority of Singapore (HSA) for the period 2010–2016 to identify various trends and signals for pharmacovigilance purposes.

## Methods

### Source of data

**Figure 2:**
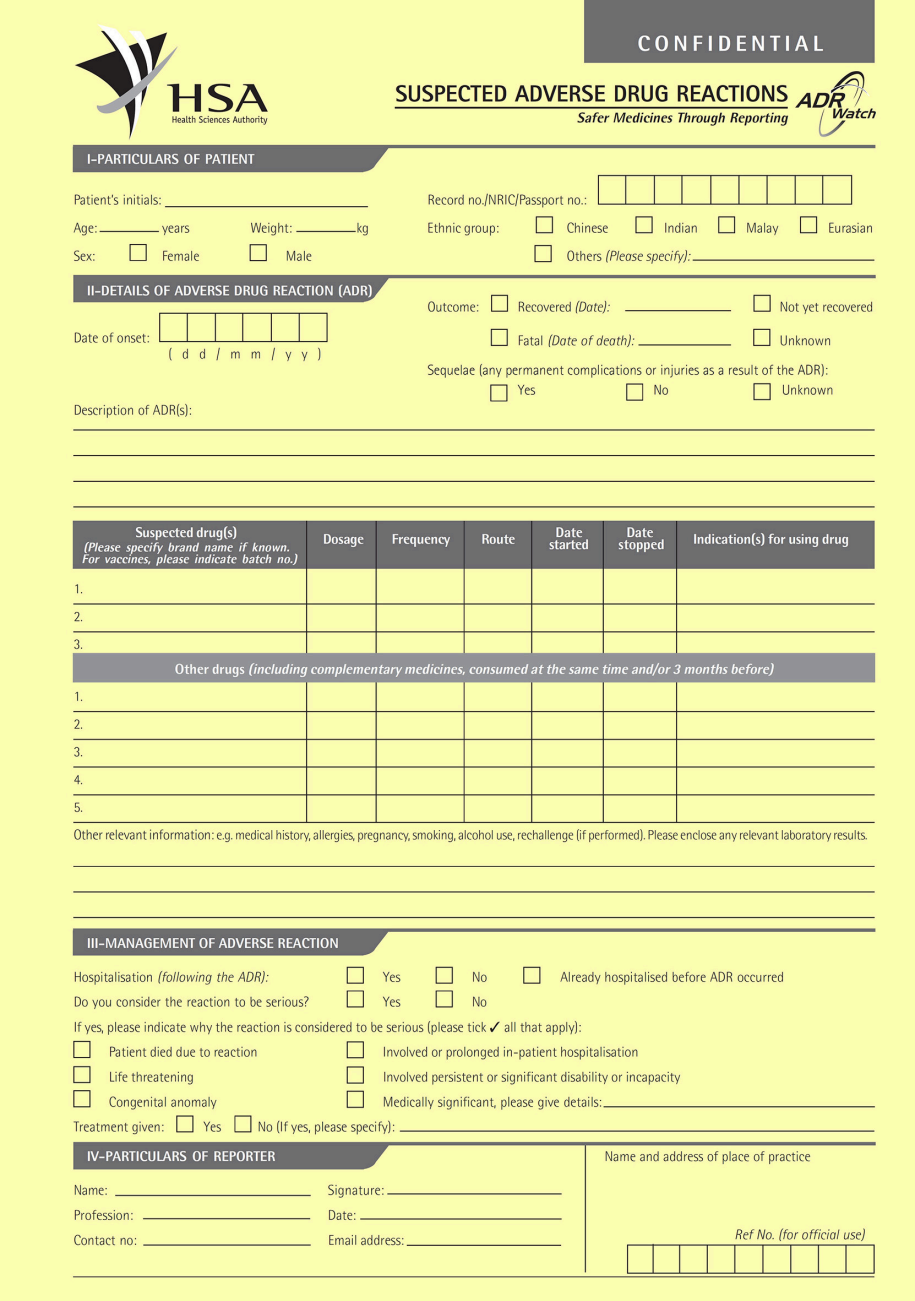
Suspected adverse drug reactions form.

The data was obtained from adverse event reports submitted to the Vigilance & Compliance Branch (VCB) of the HSA in Singapore using the “Suspected Adverse Drug Reactions” form, via e-mail, facsimile, postal mail, online or electronically through the Critical Medical Information Store component of Electronic Medical Record Exchange. The manual form is shown in Figure 2 (10). The reports were reviewed by regulatory specialists of the VCB and examined for causality of the suspected drug or herb. Further clarifications with the reporters may be conducted. All related materials and documents to each report were included in the Pharmaceutical Regulatory Information System (PRISM) database for aggregate analysis. As a member country of the WHO International Drug Monitoring Programme, these adverse event reports were also submitted to the WHO-Uppsala Monitoring Centre in Sweden for collation into WHO's VigiLyze (11).

### Data collation and analyses

The data used for analysis was for the period between January 2010 and December 2016 and was generated on 19th January 2017. The analysis was performed on valid reports that were approved and with VCB assessed causality terms “certain,” “probable,” and “possible.” To be a valid report, the minimum information required includes the following: an identifiable reporter/healthcare professional, an identifiable patient, an adverse event and a suspected product. The focus of the study was on CHP, namely CPM, health supplements and other types of complementary medicines. Information to be used in the study was extracted from the reports and collated on Microsoft® Excel files. Patient's demographic information such as age, gender, ethnicity, hospitalization status, and outcome of the adverse event, seriousness of the adverse event, organ system affected, profession of the reporter, type and description of products involved, route of administration, organ system affected, concomitant use of conventional drugs, testing for adulteration, and test results were collated.

Adverse events in the reports were categorized using the WHO adverse reaction terminology (WHO-ART) involving different system-organ classes (SOC) (12). Hospitalization status of the patients was categorized into “hospitalized,” “not hospitalized,” and “already hospitalized.” The term “already hospitalized” describes patients who have been admitted for other co-morbidities when the adverse event was detected. Outcome of the adverse events associated with the use of CHP was also analyzed by categorizing them into four categories, namely, “patients who recovered,” “not yet recovered at the point of reporting,” “had uncertain outcome,” and “died.” Conventional medicines consumed by the patient at the same time or within 3 months at the time of reporting were considered as concomitant medication. To ascertain the likelihood of the cause of the adverse event, some implicated products were sent to the Pharmaceutical Laboratory of HSA to test for the presence of drug adulterants and toxic heavy metals. Test results of those products were also collated. Information on the type and frequency of adulterants was compiled for products found to be adulterated. The variables collected from the reports were analyzed using descriptive statistics.

## Results

### Characteristics of the adverse event reports

A total of 147,215 adverse event reports suspected to be associated with pharmaceutical products and CHP were received by HSA between 2010 and 2016. Of these, 143,191 (97.3%) were associated with chemical drugs, 1,807 (1.2%) with vaccines, 1,324 (0.9%) with biological drugs (biologics), and 893 (0.6%) with CHP. Each adverse event report may involve more than 1 suspected drug or product type, more than one adverse event and more than one system organ class. The CHP surveyed were CPM, other types of complementary medicines and health supplements. Table 1 shows the number of adverse event reports associated with different CHP for the period from 2010 to 2016. Four hundred and forty nine out of the 893 (50%) cases were found to be serious according to the assessment of the VCB including 14 deaths.

**Table 1:** Number of adverse event reports associated with different types of complementary health products for the period 2010–2016.

**Year**			Number of adverse event reports associated with different types of complementary health products^*^		
	**Total no. of adverse event reports associated with pharmaceutical products and complementary health products**	**Total no. of adverse event reports associated with complementary health products**	**Chinese proprietary medicine**	**Other types of complementary medicine**	**Health supplements**
2010	23,143	99	10	23	67
2011	22,749	131	10	44	79
2012	21,828	134	9	39	89
2013	19,009	117	18	37	63
2014	19,223	135	8	25	105
2015	19,886	124	9	18	100
2016	21,377	153	8	24	122
Total	147,215	893	72	210	625

* *There may be more than 1 product type, adverse event or system organ class involved in each adverse event report*.

Among the 625 adverse event reports associated with health supplements, 517 (82.7%) were associated with products containing glucosamine and were mostly non-serious hypersensitivity reactions. Three hundred and seventy five of these cases involved female patients, 136 cases involved male patients and 6 cases did not report the gender of the patients. Three hundred and twenty nine cases related to glucosamine indicated that the adverse event was an allergic reaction while 43 cases were deemed not allergic reactions. In 140 cases, it was uncertain whether the adverse event was an allergic reaction while 5 cases did not provide the relevant information.

CPM (72 cases) and other types of complementary medicine (210 cases, out of which 76 were related to Chinese herbal remedies) were also implicated in the adverse events. Only 1 out of 72 CPM was found to be adulterated with undeclared drugs. However, 41 out of the 76 cases associated with other complementary medicines (related to Chinese herbal remedies) were found to be adulterated with 1 or more drugs.

### Indications and route of administration

Of the 893 cases of adverse events, only 228 (25.5%) reported the indications of the CHP implicated. Out of these 228 cases, most of the patients used the CHP to relieve pain such as joint and neck pain (75, 32.9%), for sexual performance enhancement (29, 12.7%), and for slimming purposes (22, 9.6%). A total of 270 products (32.2%) were taken orally as powders, capsules or tablets. Six products were used topically. The route of administration was not indicated for the remaining 617 (69.0%) cases. Eighty-six patients (9.6%) took both CHP and concomitant conventional drugs during the study period. For the remaining 807 reports, there was no information on whether the patients were taking any concomitant medicine.

### Hospitalization and outcomes of the adverse events

One hundred and fifty four (17.2%) patients were hospitalized due to the adverse events. Ninety-one (10.2%) patients were not hospitalized and 15 (1.7%) patients were already hospitalized at the point of reporting. However, information on hospitalization was not available for 633 (70.9%) cases. Further analysis on the outcomes of the adverse events associated with the use of CHP showed that 112 (12.5%) patients had recovered at the time the reports were made, 120 (13.4%) patients were not yet recovered, 607 (68.0%) had uncertain outcome and 14 died (1.57%). There was no mention of the outcome for the remaining 40 cases. Five out of the 14 fatal cases were associated with the adulteration of CHP with undeclared drugs.

### Demographic data, profession of reporters, and source of reporting

Table 2 shows the patient demographic data, profession of reporters and source of reporting. In the reports where the gender was known, 66.4% (593 cases) involved females and 32.5% (290 cases) involved males. Most of the patients were Chinese (72.8%), followed by Malays (11.2%), and Indians (6.5%). Majority of the reports were contributed by the public hospitals/healthcare organizations and polyclinics (89.8%). The remaining reports were submitted by private hospitals/clinics (8.4%), retail pharmacies (0.3%), pharmaceutical companies (1.1%), and others (0.3%). Reports were submitted mainly by doctors (86.9%) and pharmacists (10.3%).

**Table 2:** Patient demographic data, profession of reporter, and source of reporting of a total of 893 adverse event reports.

**Demographic data of patients**	**No. of cases, *n* (%)**	**Reporting source**	**No. of cases, *n* (%)**
**Gender**		**Profession of reporter**	
Female	593 (66.4)	Doctor	776 (86.9)
Male	290 (32.5)	Pharmacist	92 (10.3)
Unknown	10 (1.1)	TCM practitioner	8 (0.9)
		Manufacturer	10 (1.1)
**Age**		Nurse	4 (0.5)
<1	1 (0.1)	Others	3 (0.3)
1–20	34 (3.8)		
21–40	87 (9.7)	**Source of reporting**	
41–60	320 (35.8)	Public hospitals /polyclinics	802 (89.8)
>60	429 (48.0)	Private hospitals/clinics	75 (8.4)
Unknown	22 (2.6)	Pharmaceutical companies	10 (1.1)
		Retail pharmacies, others	6 (0.6)
**Ethnicity**			
Chinese	650 (72.8)		
Malay	100 (11.2)		
Indian	58 (6.5)		
Others	31 (3.5)		
Unknown	54 (6.0)		

### Adverse events grouped according to system organ class (SOC) classification

Table 3 shows the SOC classification of adverse events associated with the use of CHP. From the 893 reports, 1,111 different adverse events were reported. The two major SOC reported were “skin and appendages disorders,” followed by “body as a whole—general disorders” e.g., anaphylactic reaction, malaise, fever, and pain. Of these, 402 (44.9%) reports were hypersensitivity reactions e.g., rash, pruritus, and periorbital oedema. Serious adverse events during the study period included Cushing's Syndrome (36 reports), Stevens-Johnson Syndrome (5 reports), acute renal failure (6 reports), hepatic failure (11 reports), severe hypoglycemia with coma (6 reports), toxic epidermal necrolysis (3 reports), stroke (2 reports), anaphylaxis (1 report), and exfoliative dermatitis (1 report). Twenty-nine out of the 36 reports with patients experiencing Cushing's Syndrome involved adulteration of products with drugs, in particular steroids. All six reports of severe hypoglycaemia with coma shock involved sexual performance enhancement products adulterated with glibenclamide and sildenafil. Three cases of liver failure were associated with products adulterated with undeclared drugs (chlorpheniramine, dexamethasone, and paracetamol) and one of these products was also found to contain arsenic at almost 25 times above Singapore legal permissible limits of 5 ppm.

**Table 3:** Adverse events associated with the use of complementary health products classified by System Organ Class (SOC) according to the WHO Adverse Reaction Terminology (WHO-ART) arranged in decreasing order of prevalence.

**No**.	**SOC involved**	n^*^
1.	Skin and appendages disorders	502
2.	Body as a whole—general disorders	130
3.	Metabolic and nutritional disorders	71
4.	Liver and biliary system disorders	70
5.	Central and peripheral nervous system disorders	53
6.	Gastro-intestinal system disorders	51
7.	Endocrine disorders	45
8.	Urinary system disorders	32
9.	Psychiatric disorders	26
10.	Respiratory system disorders	24
11.	Vascular (extracardiac) disorders	22
12.	Musculo-skeletal system disorders	21
13.	Heart rate and rhythm disorders	15
14.	Platelet, bleeding and clotting disorders	12
14.	Cardiovascular disorders, general	11
16.	Vision disorders	5
17.	Myo-, endo-, and peripheral and valve disorders	4
18.	White cells and reticuloendothelial system disorders	3
19.	Application site disorders	2
20.	Red blood cells disorders	2
21.	Reproductive disorders, female	2
22.	Hearing and vestibular disorders	1
23.	Others	7
	Total	1,111

* *Number of cases reported in each system organ class*.

### Adulterants

One hundred and sixty (17.9%) products out of the 893 CHP were sent for laboratory testing during the study period from 2010 to 2016. Eighty-five out of 160 (53.1%) products were found to be adulterated with undeclared drugs. These adulterated products consisted of 1 CPM, 3 health supplements and 81 other types of complementary medicines. Common drug adulterants were not detected in the remaining tested products. The other 734 products were not sent for testing due to various reasons, such as unavailability of the products and absence of strong causation between the adverse events and the products. As can be seen from Table 4, the top 3 drug adulterants found are dexamethasone, chlorpheniramine, and piroxicam. In our study, 63 out of 85 cases associated with adulterated products were found to involve drugs used to alleviate pain.

**Table 4:** Adulterants (in alphabetical order) detected in complementary health products, with the frequencies of the 3 most commonly detected drug adulterants in bold.

**Adulterants**	Frequency^*^
Betamethasone	2
Betamethasone-17-valerate	1
Cetirizine	1
Chloramphenicol	1
Ciprofloxacin	1
Chlorpheniramine	**31**
Cyproheptadine	3
Dexchlorpheniramine	2
Dexamethasone	**43**
Dexamethasone Phosphate	1
Dextromethorphan	1
Diclofenac	2
Dicyclomine	1
Ephedrine	1
Famotidine	1
Furosemide	8
Glibenclamide	4
Hydrochlorothiazide	3
Ibuprofen	1
Indomethacin	2
Ketoconazole	1
Oxetacaine	2
Paracetamol	8
Pheniramine	1
Phenolphthalein	1
Phenylbutazone	4
Piroxicam	**12**
Prednisolone	1
Prednisone	3
Sibutramine and analogs	5
Sildenafil, other PDE-5 inhibitors and analogs	8
Sulphamethoxazole	1
Terbinafine	1
Tetracycline	1
Thiamine	1

* *A product could be adulterated with more than 1 adulterant*.

As shown in Table 5, 48 out of 85 adulterated products were found to contain more than 1 adulterant. These include “LifeSparks 100% Natural Pain Relief Supplement,” a health product which was found to contain 6 drugs, namely chlorpheniramine, dexamethasone, diclofenac, paracetamol, sulphamethoxazole, and piroxicam (13). Another adulterated product called “Herbal Health Jointcare,” was found to be adulterated with betamethasone-17-valerate, piroxicam, furosemide, chlorpheniramine, and famotidine (14).

**Table 5:** Frequency of complementary health products with different number of undeclared drug adulterants detected.

**No. of adulterants detected**	**No. of products (*n* = 85)**
6	1
5	4
4	3
3	16
2	24
1	37

### Toxic heavy metals

In this study, 2 cases involved products with high arsenic content. In one case, a patient had consumed a complementary medicine found to contain 120 ppm of arsenic. The patient had acute liver failure and subsequently died. The coroner's report stated that the cause of death was “multi-organ failure, following liver failure consistent with being due to auto-immune hepatitis. Death was consistent with being due to a natural disease process.” In addition, the report stated that based on the urine test, there was no evidence to suggest acute arsenic poisoning. In another case, a mother reported dramatic improvement in her young child's chronic eczema shortly after applying “TCM Recipe Licozen Ointment” (15), prompting the attending doctor to alert HSA. The product claimed to cure skin disorder and was natural, free of steroids and safe for use. High concentration of arsenic was detected in the product and a press release was issued to alert the public to stop using the potentially harmful product.

## Discussion

### Characteristics of the adverse event reports

This study presents and analyses the pattern of adverse event reports associated with the use of CHP in Singapore during a 7-year period from January 2010 to December 2016. The total number of adverse event reports in Singapore has grown over the years, with a significant increase in 2006 (1,171 reports in 2005 vs. 11,984 reports in 2006) (16, 17) as that year corresponded to the first electronic submission of adverse event reports via the Critical Medical Information Store. Adverse event reporting remains one of the most important ways of monitoring the safety of a health product throughout its marketed life. Singapore has one of the highest number of valid reports per million inhabitants that was submitted to the WHO global pharmacovigilance database from 2010 to 2015 according to the reports published by WHO's Uppsala Monitoring Centre (18).

During the 7-year study period, there was significantly higher number of adverse event reports associated with health supplement products (625 reports) compared to the preceding 12-year period study with 102 reports (19, 20). This may be due to the increasing popularity of health supplements in Singapore over the years, and more importantly, the inclusion of non-serious adverse effect terms (e.g., rash, watery eyes) into the PRISM database with effect from September 2010 (21).

Most of the reports were associated with products containing glucosamine. It might be due to the prevalent usage of glucosamine in public institutions such as the polyclinics and public hospitals. HSA receives adverse event reports mainly from these institutions. Glucosamine health supplements are commonly used for the treatment of osteoarthritis and are generally safe for use (22). Nevertheless, there are still concerns about their potential to cause allergies (23). Glucosamine is available as the sulfate or hydrochloride salt. Glucosamine sulfate used in health supplements is commonly derived from the shells of crustaceans, hence the possibility of contamination with allergens, such as tropomyosin if proper purification is not carried out (24). Tropomyosin is a muscle protein and is one of the major allergens responsible for ingestion-related allergic reactions due to shellfish (25). Hence, it is important for glucosamine products to indicate the presence of shellfish if it is part of the ingredients as most of the 517 reports were reported as allergies to the suspected products. Furthermore, dietary supplements that contain glucosamine often contain additional ingredients such as chondroitin sulfate and methylsulfonylmethane. Although chondroitin sulfate and methylsulfonylmethane have good safety profiles with very few known and mild side effects (26, 27), mild skin and eye irritation have been observed when methylsulfonylmethane is applied topically (28). Gastrointestinal discomfort has been reported with chondroitin sulfate (29).

A number of CPM and other types of complementary medicines related to Chinese herbal remedies were also implicated in the adverse events. This may be due to higher usage of such products by the Chinese population which comprised about 74% of the total population of Singapore (30) and the increasing trend of seeking TCM treatment by other non-Chinese ethnic groups (31).

### Demographic data, profession of reporters, and source of reporting

#### Age

In this study, almost half of the cases involved patients above the age of 60 and most of the implicated products were for the purpose of relieving and controlling back, joint and neck pain. This could be explained by the correlation between aging and prevalence of pain. About 19.5% of the participants in an epidemiological study of older adults (age 60 and above) experienced pain in the past month (32). In another study, 8.7% of the interviewed adults (age 18–65) had chronic pain issues, and the major cause of pain was due to musculoskeletal conditions, e.g., arthritis (33). Many elderly tend to dismiss joint pain and body aches as part of aging and would rather endure the pain or self-medicate by taking CHP that were marketed for pain relief with the perception that they were natural and safe. It had also been observed in other studies that the incidence of adverse events among older adults were significantly higher than other age groups (34). Older patients are more susceptible to adverse reactions due to comorbid conditions, polypharmacy and sensitivity to drug effects (35). Older people are also more likely to have various health problems including the decreasing functions of liver and kidney (36). Altered drug metabolism and pharmacokinetics may result in the drugs remaining longer in an old person's body, thus prolonging the drug effects and increasing the risk of side effects (37). Furthermore, drugs are also less likely to be studied extensively in the very young and the elderly.

#### Gender distribution

There were more adverse events involving females than males. The results were similar to other reports (38–40). One plausible explanation for this observation could be due to the higher usage of CHP by women than men as most of the cases reported were associated with health supplements containing glucosamine. Gender related differences in the frequencies of adverse event reporting may be due to various reasons including (i) pharmacokinetic or pharmacodynamics factors, (ii) polypharmacy, and (iii) differences in reporting patterns. Higher preference for CHP in women had also been reported in both western and eastern countries (41, 42). The prevalence of glucosamine use among women was 60.5% in an Australian study involving 266,844 participants aged 45 and above (43). Women are more likely to consume glucosamine than men due to musculoskeletal pain (44).

In addition, the higher incidence of adverse events among women may be due to anatomical and physiological differences between females and males. Women have lower bodyweight and organ size, higher body fat, slower gastric emptying time, and slower organ blood flow (45). These differences can affect the absorption, distribution, metabolism and elimination of drugs (46). For example, the higher proportion of body fat in women and slower organ blood flow may result in faster onset of action and prolonged duration of certain lipid-soluble drugs (47). Thus gender factor is expected to play a significant role in the incidence and severity of drug reaction.

Studies had shown that the hepatic enzyme CYP3A4 is more active in females than males, leading to differences in the drug metabolism in the two genders (48). For example, the metabolism of midazolam in women is more than that in men due to the activity of CYP3A4 (49).

Pronounced differences between women and men were seen in a study on the incidence of adverse events caused by cardiovascular drugs (50). In that nationwide investigation, women were found to be more frequently admitted with an adverse event related to high-ceiling diuretics, low-ceiling diuretics, and cardiotoxic glycosides than men. However, adverse event-related admissions associated with coronary vasodilators were more frequently reported in men. Pharmacodynamic differences between men and woman are particularly seen with cardiac drugs. For example, aldosterone is directly related to cardiac wall thickness in women but not in men, suggesting that women are more at risk from the harmful effects of aldosterone (51). Another plausible reason for the gender distribution is that women are more willing to report adverse events in contrast to men (52).

### System organ class (SOC) classification

As can be seen from Table 3, majority of the adverse events were classified as “skin and appendages disorders” and the clinical manifestation was mainly rashes. Based on the data, reports involving products containing glucosamine could be due to allergy to possible allergens (e.g., tropomyosin) in the products as discussed earlier in the section Characteristics of the Adverse Event Reports. Further analysis was not possible as the total ingredient listings of each product had not been included in the reports. In addition, 19 adverse events were associated with *Ginkgo biloba*. Extracts of *G. biloba* leaf are used to treat various health problems such as dementia, memory deficits, headaches, intermittent claudication, vertigo, and tinnitus (53, 54). *Ginkgo Folium* (Ginkgo leaf, 银杏叶) and *Ginkgo Semen* (Ginkgo seed, 白果) are also used in TCM and Japanese Kampo. *Ginkgo Folium* in TCM is used to “activate the blood, resolve stasis, unblock the collaterals, relieve pain, astringe the lung, relieve wheezing, resolve turbidity, and lower lipid” (Committee of National Pharmacopeia of PR China 2015). *Ginkgo Semen* is used for the treatment of profuse sputum, wheezing and cough, vaginal discharge with white turbidity, enuresis and frequent urination (Committee of National Pharmacopeia of PR China 2015). In recent years, *G. biloba* has become a popular herbal supplement marketed to improve age-related physical and cognitive disorders (55). However, due to the presence of trace amounts of ginkgolic acid in the leaves and nuts, *G. biloba* may provoke allergic reactions (56, 57).

“Body as a whole—general disorders” was found to be the second most commonly reported SOC in the current study. This involves hypersensitivity, periorbital oedema, chest/back/leg pain, fatigue, and malaise etc. “Metabolism and nutritional disorders” include hypoglycaemia, hypertriglyceridaemia, and weight increase. Twenty-five out of the 71 adverse events with “metabolism and nutritional disorders” involved sexual enhancement health supplements adulterated with glibenclamide and sildenafil between the period from 2010 to 2013. These patients experienced hypoglycaemia, some were comatosed and one died.

In the WHO adverse drug reaction database, skin reactions were the most frequently reported adverse events associated with the use of herbal products. In a hospital-based observational study in Korea from April 2012 to December 2014, gastro-intestinal system disorders, followed by skin-related disorders were the most common adverse events reported in 163 herbal-drug-associated cases (58). Likewise in Sweden, the most commonly reported adverse reactions to herbal medicinal products or natural remedies were “skin and subcutaneous tissue disorders” and “gastrointestinal disorders” in a 9-year study on adverse events reported to the Swedish Medical Product Agency (59). “Skin and hypersensitivity reactions” and “gastrointestinal disorders” had also been reported as the two most common health products related adverse events (9, 60, 61).

Besides the skin and “body as a whole”, the liver was found to be one of the most affected organs in the present study. Some herbal medicines or their derived products were found to cause drug-induced liver injury (DILI). These patients experienced increased levels of hepatic enzymes, jaundice and even hepatocellular damages in serious adverse cases. Elevated liver enzymes is one of the more common adverse effects caused by herbal products (62). The National Institutes of Health has also developed a database which includes herbal medicines and dietary supplement ingredients associated with hepatotoxicity (23). Over 30 herbal medicines were reported to cause DILI. Besides prescription drugs, herbal products were ranked as the second most common cause of DILI in USA (63).

In the current study, to the best of our knowledge, no toxic herb was confirmed to be implicated in the hepatotoxic adverse events. Hepatotoxicity cases associated with herbal products on spontaneous adverse events in Singapore from 2010 to 2014 were reported (64). In that study, 35 out of the 57 adverse event reports involved hepatotoxicity, and Chai Hu (*Radix bupleuri*) was suspected in 11 of these 35 cases. Prevalence of DILI associated with CHP was reported to be 26% (98 out of 371 studied cases) in Korea (65) and 36% (130 out of 361 studied cases) in Taiwan (66). In the USA, 1,219 patients with liver injury from medications and/or dietary supplements between 2004 and 2013 showed that dietary supplement related liver injury increased from 7 to 20% during the study period (67). Furthermore, a population-based survey in Iceland reported that 16% of the 96 cases of DILI diagnosed in 2010–2011 were attributed to dietary supplements. The incidence of DILI in the population was estimated to be 19.1 per 100,000 persons (68). Due to the widespread use of herbal remedies in Asia, the percentage of DILI cases attributed to CHP in Asian countries was higher compared to the West.

Herbs which may cause DILI include *Symphytum* species (Comfrey), *Heliotropium*, and *Senecio* species (Groundsel), Germander, Chaparral, and Kava kava (69–72). Despite the benefits of green tea, green tea extract associated hepatotoxicity has also been reported (73, 74). Hepatotoxicity following the consumption of concentrated green tea extract may be attributed to the catechins due to the collapsing of the mitochondrial membrane potential of hepatocytes leading to cell death and the formation of reactive oxygen species (75). Besides intrinsic toxicity, herbal products contaminated by aflatoxins can result in acute toxic hepatitis associated with high morbidity (76). In the current study, 9 adverse event reports with liver biliary system disorders were found to be associated with CHP adulterated with various drugs. Therefore, besides reviewing the intrinsic toxicity of the herbs, it is also necessary to screen for contaminants and adulterants to assess the safety of the products.

### Adulterated health products

#### Pain relieving products

The adulteration of CHP with undeclared drugs appeared to be one of the main causes of the associated adverse events. Majority of the adulterated cases involved products labeled or marketed to treat rheumatic joint pain, backache and numbness of the limbs to relieve pain and reduce inflammation. The adulterants found were mainly non-steroidal anti-inflammatory drugs (NSAIDs) and steroids. This can be very dangerous as the usage of NSAIDs may result in cardiovascular side effects such as myocardial infarction and stroke. In addition, long term unsupervised usage of oral steroids can lead to Cushing's Syndrome, increased blood glucose levels leading to diabetes, high blood pressure, cataracts, muscular and bone disorders, and an increased risk of infections (77, 78).

Dexamethasone is a potent steroid usually prescribed for inflammatory conditions and used under strict medical supervision. Chlorpheniramine is an antihistamine which helps to reduce flu-like symptoms (e.g., watery eyes and runny nose). Piroxicam is an NSAID used for the treatment of painful inflammatory conditions e.g., arthritis. A patient developed Cushing's Syndrome and various complications after consuming a complementary health product (Huo Li Shen Dan) adulterated with pain relief drugs, namely dexamethasone, hydrochlorothiazide, indomethacin, and prednisolone. The patient subsequently died. In a study in Hong Kong on 61 patients using proprietary Chinese medicines adulterated with corticosteroids and NSAIDs from 2008 to 2012 (79), 11.5% of the patients required intensive care and 2 died within 30 days of presentation.

#### Slimming products

Products used for slimming purposes were also found to be adulterated with synthetic drugs such as sibutramine, benzyl sibutramine, and phenolphthalein. Sibutramine, a prescription medicine previously used in the treatment of exogenous obesity, had been banned for sale in the EU, USA and Singapore markets since 2010 because of safety concerns about its cardiovascular risks (80, 81). The use of sibutramine may cause serious adverse effects, including tachycardia, hypertension, anxiety, heart attacks, and irregular heartbeats. However, the drug continues to be fraudulently added to weight loss products.

According to the U.S. Food and Drug Administration (FDA) list of tainted dietary supplements, 316 out of 781 public alerts (40%) published between 2009 and 2016 involved adulterated weight loss products. Most of the cases (89%) involved the illegal addition of sibutramine (82). In another study, about half of the 52 weight loss products purchased via the Internet contained sibutramine with contents exceeding normal therapeutic dosage (83). Benzyl sibutramine, an analog of sibutramine first reported in 2013, was detected in a weight loss product “Nutri Drops Grape fruit Diet” (84–86). It was found to contain sibutramine, benzyl sibutramine, and phenolphthalein despite claiming to contain all natural ingredients. The patient experienced hallucinations and was hospitalized after consuming the product for more than 1 month to lose weight. As the safety of drug analogs is unknown, there is a potential health risk to consumers because of their structural similarity to sibutramine. Another common adulterant in weight loss products is phenolphthalein, a drug used as a laxative and has been banned due to carcinogenicity concerns. Similar to sibutramine, it is frequently listed in public notifications from the U.S. FDA.

It may be possible that the number of adverse effects of sibutramine and other drugs present as adulterants in slimming products is under reported as many patients will discontinue the use of these products when they feel unwell. The ill effects are often reversible when the adverse effects are mild.

#### Sexual performance enhancement products

In this study, some products used for sexual performance enhancement were also found to be adulterated with sildenafil (a PDE-5 inhibitor), analogs of PDE-5 inhibitors and glibenclamide. Sildenafil and glibenclamide are prescription drugs that should only be taken under the supervision of a doctor. In one of the 5 fatal cases associated with the adulteration, the patient had taken a sexual enhancement health supplement called “Power 1 Walnut,” found to be adulterated with sildenafil and glibenclamide. The patient experienced hypoglycaemia and subsequently died. The exact reasons for the presence of the anti-diabetic drug glibenclamide in sexual enhancement products are unknown but possible reasons include contamination or mixing up of ingredients during manufacturing (87). The use of such adulterated products may also be associated with side effects and drug-drug interactions as the users may have co-morbid conditions such as diabetes and cardiovascular diseases. Concomitant consumption of sildenafil and nitrate drugs may result in severe hypotension and may be fatal. More than 70 analogs of approved PDE-5 inhibitor drugs had been found to be adulterated in sexual performance enhancement products (87, 88).

Two cases in the current study involved analogs of PDE-5 inhibitors. They were N-cyclopentyl nortadalafil (89) and 3,5-dimethylpiperazinyl dithio-desmethylcarbodenafil (90). They are illegally synthesized and adulterated into herbal products to avoid detection. The USP has published a General Chapter <2251> Screening for Undeclared Drugs and Drug Analogs, which provides guidance on several screening techniques for the detection of adulterants (e.g., PDE-5 inhibitors and their analogs) (91). Future update of the chapter may include methodologies for analysis of adulterated weight loss and sports performance enhancement products. Many of these analogs are not subjected to the thorough pre-clinical and clinical studies needed for drug registration, hence their toxicities remain unknown, and they are of safety concerns to the unknowing public.

### Concomitant drug administration

The actual number of patients taking both conventional drugs and CHP may be under reported as the patients might be reluctant to share or were unaware of the importance of such information. Concurrent use of conventional drugs and CHP could result in herb-drug interactions which may lead to potentially serious adverse events. Patients most at risk of harmful drug-herb interactions are the children, the elderly, those on polypharmacy, those with chronic illnesses or impaired organ functions and those on drugs with narrow therapeutic windows such as warfarin (92). Coagulation problems arising from drug-herb interaction with warfarin had been reported, sometimes with serious consequences, such as intracranial hematoma (93). The prevalence of the disclosure of CHP usage to physicians is generally low (94–96). Fifty six percent of adult cancer patients in Singapore reported using CHP (97). However, only 54% of them informed their oncologists regarding the use of such products as most patients and caregivers do not readily disclose their use, and healthcare professionals may not routinely ask about such use. Adverse events due to drug-herb interactions may not be recognized if the physician or other health professional is not aware of the concomitant use of herbal products. Therefore, it is important for the patients and their healthcare providers to communicate openly about the usage of herbal products and pharmaceutical products.

### Toxic heavy metals

Toxic heavy metals are of special health concerns due to their potential toxic effects. Arsenic and mercury can result in increased risk of various disorders such as cardiovascular abnormalities (98, 99), neurotoxicity, hepatotoxicity, nephrotoxicity, and carcinogenicity (100, 101). Cadmium toxicity has been demonstrated in several organs, especially the kidney. It can induce tissue injury through creating oxidative stress, changes in DNA expression and inhibition or upregulation of transport pathway particularly in the proximal segment of the kidney tubule (102). Chronic exposure to lead may cause adverse effects on the central nervous system, blood pressure, kidneys, and vitamin D metabolism mainly through lead's ability to inhibit or mimic the actions of calcium and to interact with proteins (101). Excessive intake of copper can lead to cellular toxicity through free radical-induced oxidative damage. Copper toxicity has been associated with Wilson's disease, renal and hepatic failure when copper homeostasis is disrupted (103).

### Regulatory actions

HSA has in place a product quality surveillance programme to conduct risk-based sampling and testing of CHP on the market. Manufacturers and importers of CHP must ensure that their products are free of undeclared drugs and drug analogs or toxic heavy metals above the permissible legal limits. When adulteration or non-compliance to heavy metal limits have been established, HSA will issue press releases, alert healthcare professionals and/or take actions to remove the affected products from the market.

## Limitations

As the study is based on the retrospective analysis of spontaneous reports of adverse events, certain information such as indications of products, route of administration, concomitant drug administration, and other relevant information regarding the patients' medications were often lacking in the reports. In addition, patients might have been unwilling to share certain information that they deemed sensitive. Also, in many cases, the exact herbal ingredients were not described in the report or a sample of the complementary health product was not available for testing. Therefore, without performing further toxicity tests on the products, it was not possible to determine whether the suspected products might contain herbal ingredients with toxic constituents. In addition, the use of concomitant drugs and the presence of adulterants (e.g., undeclared drugs and drug analogs) in the products could also lead to adverse events (19). Likewise, individual patient's medical conditions may predispose one to adverse events. The classification of an adverse event as being due to a complementary health product (i.e., decision with regards to causality) depends on the reporter's account and assessment, besides the presence of adulterants and toxic heavy metals. If the product is available, it may be sent to HSA laboratories for analysis. Manufacturers are usually unaware of the adverse event or the products may be illegal. The adverse events are usually observed by the healthcare professionals when patients seek treatment and then reported by the healthcare professionals as their ethical duty. The information whether both pharmaceutical products and CHP are used concomitantly may not be present in the adverse event reports. In such situations, there may or may not be concomitant usage of both. Hence, a causal relationship cannot be confidently drawn. Moreover, under-reporting (104) might occur due to various plausible reasons, e.g., (1) patients do not inform their healthcare providers on their usage of CHP, (2) lack of association between the product and adverse effects, (3) mild self-limiting adverse effects upon stopping the use, (4) sensitivity of information (e.g., with slimming and sexual performance enhancement products), (5) healthcare professionals are unaware that herbal adverse events should be reported, and (6) patients may not consider herbal and nutritional products to be “medicines” and hence neither disclose the use nor seek professional advice before using them as most of the products are available over-the-counter. Under reporting is a challenge with spontaneous reporting system (105). Since large post market surveillance studies of herbal medicine will require extensive resources, spontaneous reporting of adverse event is still an essential and effective pharmacovigilance tool. Currently, the Health Products Act in Singapore legislatively mandates the reporting of serious adverse events by the manufacturer, importer, supplier or registrant of therapeutic products (pharmaceutical products), medical devices and cosmetic products. All local manufacturing facilities engaged in the manufacture or assembly of pharmaceutical products and CPM must be licensed with HSA. The manufacturers are expected to comply with the relevant legislative and regulatory requirements, and GMP standard. For imported CPMs, GMP from country of origin is required where applicable (i.e., if imposed by country of origin). Local manufacturers of CPMs must be licensed with HSA.

In the United States, most herbal products are classified under Dietary supplements and regulated under the Dietary Supplement Health and Education Act (DSHEA) 1994 (106). In Australia, medicinal products containing such ingredients as herbs, vitamins, minerals, nutritional supplements, homeopathic, and certain aromatherapy preparations are referred to as “complementary medicines” and are regulated as medicines under the Therapeutic Goods Act 1989 (107). It is beyond the scope of this paper to compare the classification system in Singapore and Western countries. Regardless of classifications, there is no distinction between the way adverse events due to different products (e.g., pharmaceutical products and CHP) are handled. The adverse event reports are managed in the same database and based on botanical names where possible.

## Conclusion

In conclusion, 893 adverse event reports associated with CHP in the period between 2010 and 2016 have been successfully collated and analyzed. They constituted about 0.6% of the total number of adverse events reported. The majority of the adverse event cases in that period were still due to conventional drugs. In agreement with other studies, adverse reactions related to the “skin and appendages disorders” were the most commonly reported reactions. Most of the cases involved the use of glucosamine products and most of the adulterated products were associated with the illegal addition of drugs for pain relief. Even if the causality is not confirmed, reporting of suspected adverse events is strongly encouraged as rapid regulatory actions can be taken if there is any sign of clustering. The findings from this study help to create greater awareness on the health risks, albeit low, when consuming CHP and dispelling the common misconception that “natural” means “safe.” In particular, healthcare professionals and the general public should be aware of potential adulteration of CHP. The analysis of spontaneously reported adverse events is an important surveillance system in monitoring the safety of CHP and helps in the understanding of the risk associated with the use of such products. Greater collaboration and communication between healthcare professionals, regulators, patients, manufacturers, researchers, and the general public are important to ensure the quality and safety of CHP, while harnessing their potential benefits.

## Author contributions

YX collated the data, analyzed the data and drafted the manuscript; DP, S-LN, S-HT, DT, JP, and ATL corrected the manuscript; C-LC, M-YL, and H-LK conceptualized the project and corrected the manuscript.

### Conflict of interest statement

The authors declare that the research was conducted in the absence of any commercial or financial relationships that could be construed as a potential conflict of interest.
